# Factors that affect pathways to care for youth with psychotic disorders in Newfoundland and Labrador, Canada: A qualitative study

**DOI:** 10.1371/journal.pmen.0000612

**Published:** 2026-05-14

**Authors:** Zachary E.M. Giovannini-Green, Gerald Mugford, David Philpott

**Affiliations:** 1 Division of Population Health and Applied Health Sciences, Faculty of Medicine, Memorial University of Newfoundland, St. John’s, Newfoundland and Labrador, Canada; 2 Faculty of Education, Memorial University of Newfoundland, St. John’s, Newfoundland and Labrador, Canada; UTHSC: The University of Texas Health Science Center at Houston, UNITED STATES OF AMERICA

## Abstract

Youth mental health has become an area of concern in Newfoundland and Labrador (NL), with psychotic disorders representing some of the most disabling forms of mental illness. Yet the pathways to care (PtC) that youth in NL use to access mental health supports remain poorly understood. This research used a qualitative approach to examine the experiences of this population in three important areas: 1) What patterns emerge in the early symptoms and behaviours of children who develop a psychotic disorder? 2) What factors influence treatment and diagnosis from the perspectives of clients and families? 3) What gaps exist in services for clients in NL living with a psychotic disorder and their families? Using a grounded theory approach, this study employed semi-structured interviews and focus groups across two phases. Phase one included participants who were: 1) clients of mental health services, nineteen or older, diagnosed with a psychotic disorder between ages 15–24, and 2) a close family member most knowledgeable about the client’s history. Phase two involved interviews with caseworkers, whose professional experience added valuable context and enabled triangulation of findings. The data from this study informed the creation of categories, which developed into the concepts of the grounded theory. First, the study documented clients’ developmental histories and changes that appeared with the onset of symptoms. Second, it highlighted the challenges clients and families encountered in obtaining a diagnosis, managing medications, and accessing sufficient community support. Third, it identified gaps in services from the perspectives of clients and family members, along with their recommendations for improvement. Caseworkers recognized these themes as consistent with their own experiences working with affected youth and families. This study amplifies the voices of clients and family members to illustrate PtC for NL youth living with psychotic disorders. It is intended as a resource for clinicians, caseworkers, school personnel, and policymakers to support future policy development that strengthens services, enhances care pathways, and improves outcomes for this vulnerable population.

## Introduction

### Background

The transition from childhood to adulthood can be difficult in the most opportune circumstances without the influence of major medical complications. Even with the support of the community, this transition becomes exponentially more difficult when symptoms of psychotic illness appear, causing struggles which negatively affect health outcomes. Examining the pathways to care (PtC) that youth and their families use to access diagnosis and treatment has become a formalized area of inquiry. The primary source of data in this research is: 1) clients of mental health services diagnosed with a psychotic illness between the ages of 15 and 24, and 2) family members of those clients. This necessitated a qualitative approach to the data collection and the analysis. Interviews were conducted with both clients and family members to collect experiences and perspectives on their lived experiences with PtC. To help triangulate the data, caseworkers in psychosis management were recruited for focus groups to provide their insights and experiences. What has emerged provides valuable insight into the patterns that form the PtC for this cohort, as well as early indicators that could help inform service providers.

### Literature review

A recent study by The Lancet Psychiatry identifies that the burden of mental illness has been rising globally, with some of the highest effects being felt in North America [1]. Each year in Canada, approximately 18% of our population will reach the clinical criteria to be diagnosed with at least one mental illness [2]. Schizophrenia, the most debilitating of the psychotic disorders, affects approximately one percent of Canadians [3] and 0.3% of the global population [4]. A recent study by Myran et al. [5] showed that the incidence of psychotic disorders in Canadian youth aged 14–20 increased by 60% from 1997 to 2023.

In Newfoundland and Labrador (NL), a recent provincial health review [6] reported mental health as being “consistently identified” among the most serious concerns of the province’s youth during town halls. Newfoundlanders and Labradorians must wait on average longer than other Canadians for mental health services. In the 2020 fiscal year, the average wait time for community mental health counselling in NL was 33 days, 11 days longer than the national average. Wait times peaked in the 2022 fiscal year at 67 days, more than double the national average of 31 days, before decreasing to 44 days in the 2024 fiscal year (14 days above the national average) [7]. The provision of community mental health services in NL is complicated by a rural population more than twice the national average (40.0% versus 17.8%) [8]. Furthermore, mental health professionals, including psychologists, have been leaving the public health sector for the private sector, creating significant vacancies [9]. The delays in mental health services caused by these factors can increase the severity of their symptoms and, in turn, increase the cost of adequately treating them.

Wait times are particularly problematic for youth transitioning from child to adult health services. The peak age of onset of schizophrenia and related disorders occurs during this transition at approximately 20 years [10]. The onset of acute psychosis is often preceded by an early phase of psychosis known as the prodromal phase [11], usually beginning in adolescence. The onset of the prodromal phase will add additional stressors to youth during an already difficult stage in their development. Symptoms cause distress to both clients and family members and include irritation, anxiety, disturbed sleep, decreased ability to concentrate, withdrawal, academic disruption, and the development of bizarre thoughts and ideas [12]. Stigma, both from others and from themselves, has been shown to delay accessing treatment and prolong the duration of untreated psychosis [13]. The duration of untreated psychosis (DUP) is accepted as a significant predictor of outcomes for those who experience psychotic episodes [14,15]. This is especially relevant for youth exiting a relatively supportive school environment as they transition from pediatric to adult healthcare.

The effects of these symptoms greatly affect the ability of young people to access help for their mental health. In Canada, only 20–25% of youth will seek out any form of support for their mental health, and when they do, it is often from online resources versus health professionals [16,17]. Canadian research recommends increased mental health services for youth, given the rise in prevalence in recent years [18]. In an orchestrated push to understand how health care is sought, the term pathways to care (PtC) was formalized in the 1990s [19]. PtC has since become a significant area of study to understand the contacts a patient and their family make to access healthcare services, often identifying inequalities in access to those services [20–22].

### Rationale and research objective

Healthcare is predominantly the responsibility of the provincial government, with the federal government setting guiding principles and providing funding [23]. The PtC for individuals living with psychosis and their families, therefore, differ across Canada due to diversity in the structure and administration of health care systems [24]. As far as these authors are aware, this is the first study in NL that has specifically sought to: 1) describe patterns of symptoms and behaviours in childhood and 2) comprehensively describe the gaps in service for this population. Therefore, a research design was selected and implemented, which described the experiences of people diagnosed as youth with a psychotic disorder in NL in relation to: 1) the patterns in behaviours and experiences that describe earlier development and the first stages of psychosis, 2) the factors associated with the diagnosis and treatment of youth with a psychotic disorder in NL, and 3) existing gaps in services for psychosis care in NL youth.

## Methods

### Ethics statement

Ethical approval for this study was granted by the Health Research Ethics Authority of Newfoundland and Labrador (HREB #2022.214). Informed consent was obtained in writing from all study participants

### Grounded theory

Answering these questions required that data be collected from participants to inform a coherent theme, which captured the PtC for clients and families in NL. A grounded theory approach was well-suited to the inductive logic required in this research. Grounded theory is often used when little is known about a topic of research and when an explanatory and descriptive theory is required [25]. It was first developed by sociologists Glaser and Strauss in the 1960s [26] and has continued to grow and evolve since that time [25,27]. The grounded theory approach involves a “systematic method of conducting research that shapes collecting data and provides explicit strategies for analyzing them” [28] and refers to both the method of conducting research and the product of the method [27].

This study employed constructivist grounded theory as developed by Kathy Charmaz [29,30]. Constructivist grounded theory outlines a specific and useful approach to applying grounded theory. Charmaz identifies four key criteria [29,30] that are essential to a grounded theory and were used as touchstones for this study to ensure rigour: credibility, originality, resonance, and usefulness. Credibility in constructivist grounded theory requires having adequate data relevant to the study to perform the analysis, as well as remaining aware of and examining one’s own perspectives and biases. Originality refers to the purpose of grounded theory to offer new insights into a problem. Resonance in constructivist grounded theory describes both how the data capture participants’ experiences and how those experiences are reported to readers. Finally, the usefulness of a grounded theory depends on whether it: 1) illuminates processes in participants’ lives, 2) opens new lines of research, and 3) informs the development of new policies and protocols grounded in the theory.

### Data collection

The project sought to understand the perspectives of clients and family members as they pertained to receiving a diagnosis and treatment of a psychotic disorder. The methodology employed had to be flexible to accommodate individual insights and perspectives while remaining consistent across interviews. To achieve this, semi-structured interviews and focus groups were selected to gather this data, and purposive sampling was used to recruit key informants. All participants were informed of their right to pause the interview or focus group or withdraw from the study at any time. This occurred both during the consent process and at the beginning of the interview or focus group.

The interviews and focus groups took place between July 26^th^, 2023, and June 21^st^, 2024. Webex, a virtual conferencing service, was used to conduct all but one of the caseworkers’ focus groups and thirteen of the fifteen client and family member interviews. Two interviews took place in a meeting room of the Waterford Hospital, and one caseworker focus group took place in a meeting room in Memorial University’s Faculty of Medicine building. Both buildings are located in St. John’s, Canada.

### Study participants

This study was designed to be completed in two phases. In phase one, participants recruited were: 1) clients of mental health services, nineteen years and older, diagnosed with a psychotic disorder between 15 and 24 years of age and who received treatment in Newfoundland and Labrador, and 2) a close family member who is most knowledgeable about the first participant, usually a parent. Psychotic disorders are relatively rare compared to other illnesses, and those living with them, as well as their family members, can be difficult to successfully recruit in large numbers for research projects. To obtain the most nuanced, comprehensive understanding of clients’ and family members’ experiences, a second phase was planned. Phase two of the study involved interviewing caseworkers to obtain their input on these themes, drawing from their work in the field. It should be noted that caseworker input was not intended to confirm the experiences of clients and family members, but rather to triangulate and enrich them.

Participants for this study were deliberately selected in a process called purposive sampling [31,32], whereby sources of relevant data were identified and approached. Data are then constructed and analyzed concurrently in an iterative cycle [25]. This study used an approach first pioneered by Glaser and Strauss, referred to as theoretical sampling [28,33]. Using this method, lines of inquiry that appeared in the data were investigated until data saturation was reached. This study used the definition for data saturation as described by Glaser and Strauss as the point “no additional data are being found whereby the sociologist can develop properties of the category” [26].

### Recruiting participants

During recruitment, service providers who support clients and/or families likely to meet the criteria were identified and contacted. They were then provided with a study description and participant criteria as well as the principal investigator’s contact information. If clients and/or family members in the service provider’s practice expressed interest in the study, they contacted the investigator and were presented with an informed consent document for signature. The document clearly explained the study aims, participant rights, as well as how, where, and for how long the data would be stored, as stipulated by university policy. Phase one of the study intended to conduct two separate interviews, first with the client and, with their permission, a follow-up with the family member to form a dyad. The interview with the family member was not intended to correct or modify the client’s experiences, but to obtain additional perspectives on the client’s PtC. Semi-structured interview questions were developed for participants as prompts, allowing them to share their story and share as much information as they were comfortable. Both interviews were designed to last between 45 and 60 minutes. The interview guidelines for clients and family members are provided in S1 File.

The recruitment of caseworkers followed a similar process. Caseworkers who participated in the first-phase recruitment process, as well as others in the field, were individually approached to participate in phase two. If they agreed, they were presented with an informed consent document containing the same information as discussed above for their signature. Focus groups were designed for small groups of participants and were limited to 50 minutes, allowing caseworkers sufficient time to speak without taking excessive time away from their professional duties. The principal investigator presented a series of eight statements that emerged in phase one for the caseworkers to discuss. The focus group questions based on the themes gathered from phase one of the study can be found in S2 File. Recruitment for the study began on July 3^rd^, 2023, and closed on June 23^rd^, 2024.

As expected from the beginning of this study, the vulnerable nature of those living with psychotic disorders presented difficulties in recruiting clients. Several clients agreed to participate but later withdrew. What was less expected was the impact resulting from the time commitments and stress of being a family member to clients, and the time constraints and stress of caseworkers working with clients living with psychotic disorders. These factors also led to difficulties with recruiting family members and caseworkers as participants in the study. Study recruitment took place over approximately twelve months. During this time, fifteen interviews were completed: two as a client-parent dyad, three with clients only, and ten with family members only. Thirteen families took part in the study, each with a family member who was a client of mental health services due to a psychotic disorder. Of these thirteen clients, two were female, and eleven were male. The age of the clients at the time of the study ranged from 19 years to 48 years. Time since diagnosis with a psychotic disorder ranged from three months to over twenty years. One of the thirteen clients was diagnosed as a child, while the rest were diagnosed as adults (ages 18–24). A total of seven caseworkers also consented to participate in the study, taking part in focus groups. Participant characteristics for clients and family members, as well as caseworkers, are described in Tables 1 and 2.

**Table 1:** Characteristics of Clients and Family Members.

Participant	Family	Relation to Client	Residence of Client	Client Diagnosis
Client 1	Family 1	N/A	Urban	St. John’s – APD
Family Member 2	Family 2	Parent	Rural	Out of Province
Client 3	Family 3	N/A	Rural then Urban	St. John’s – APD
Family Member 3	Family 3	Parent	Rural then Urban	St. John’s – APD
Family Member 4	Family 4	Parent	Urban	St. John’s – APD
Family Member 5	Family 5	Parent	Urban	St. John’s – APD
Client 6	Family 6	N/A	Urban	St. John’s – APD
Family Member 7	Family 7	Parent	Urban	St. John’s – APD
Family Member 8	Family 8	Relative	Urban	St. John’s – APD
Family Member 9	Family 9	Parent	Urban	St. John’s – APD
Family Member 10	Family 8	Parent	Urban	St. John’s – APD
Family Member 11	Family 10	Relative	Urban	St. John’s – CPD
Client 12	Family 11	N/A	Urban	St. John’s – APD
Family Member 13	Family 12	Parent	Urban	St. John’s – APD
Family Member 14	Family 13	Parent	Urban	St. John’s – APD

APD, adult psychiatry department; CPD, child psychiatry department.

**Table 2:** Characteristics of Caseworkers.

Caseworker	Practice Setting	Scope of Practice
Caseworker 1	Hospital	Older adults
Caseworker 2	Hospital	Clients and families
Caseworker 3	Hospital	Clients and families
Caseworker 4	Community	School-aged children
Caseworker 5	Community	Youth and families
Caseworker 6	Community	Youth and families
Caseworker 7	Community	Adults

### Analysis

Data analysis in constructivist grounded theory begins with line-by-line coding of the data [28]. As the name suggests, this involves reading every line of a text and generating codes from that text. Line-by-line coding continues until codes appear as more important, accounting for more data than other codes. These are referred to as “focused codes” and indicate that the researcher can stop line-by-line coding [28]. Focused codes are then investigated further to develop thematic categories, which are then checked against the data. Analysis in grounded theory is further aided by the practice of writing memos [34]. Memos are written records used by researchers to examine their thought processes about the analysis and identify the relationship between categories. They are also an intrinsic part of maintaining the credibility of the grounded theory as described by Charmaz [29,30] because they allow for the examination of the researcher’s own perspective and biases. The final step of the process is the integration of all categories discovered in the data to develop a potential theory or framework.

After receiving the participant’s informed consent, the interviews and focus groups were recorded using two different devices and then transcribed verbatim using the Otter.ai software. The transcribed document was then checked by the investigator by comparing it with the digital recordings to ensure accuracy. If the participant consented to be interviewed but did not consent to being recorded, the investigator made jot note recordings in a Word document.

The coding process for this study was undertaken entirely manually to ensure that the investigator was immersed in the data and attuned to any possible connections that could be developed into an explanatory theory. In phase one, the interviews with clients and family members were analyzed first. Theoretical sampling was employed to investigate lines of inquiry that appeared during the interviews until the researchers were satisfied that data saturation had been reached. Interviews with clients and family members were conducted before the focus groups with caseworkers for two main reasons. One, they were viewed as the primary source of data in this study, given their lived experience. Two, the themes uncovered in the interviews served as the basis for focus groups with caseworkers.

Line-by-line coding was used in this analysis initially to identify codes until focused codes began to appear. During this process, memoing allowed for impromptu note-taking that documented informal connections and theories that appeared as part of the analysis. The resulting focused codes were then investigated and refined to ensure that they comprehensively represented the data that was present in the interviews. The result of this step in the analysis became the themes that were used to facilitate the focus group discussions between caseworkers (available in the S2 File at the end of this article). This process was then repeated with the caseworker focus groups, with line-by-line coding leading to focused codes, which in turn produced larger categories. In the final step of the analysis, both interviews and focus groups were analyzed together. This step defined the relationships that existed between all categories, which established an explanatory narrative fully grounded in the data.

## Results

### Overview of study outcomes

The interviews with clients and family members produced several focused codes that depicted the lived experiences of these two groups. Clients and family members spoke about struggles in school beginning in the mid-teens as the first symptoms of psychosis appeared. This situation often precipitated visits to a family physician or specialist. These visits were ultimately unsuccessful in determining a diagnosis and effective treatment for the client and often left all concerned parties frustrated. In some cases, this experience led to a client’s use of recreational substances to help manage the symptoms they were experiencing. Family members recalled struggling for long periods of time before eventually finding help. Even then, they continued to struggle to manage the symptoms of their loved one’s illness, with some family members being forced to call the police to deal with especially challenging behaviours. Accessing diagnosis and treatment for clients was an even more difficult and lengthy process in rural parts of the province, where resources are less available. In particular, the appropriate management of medications and their adverse effects repeatedly appeared as an area of serious concern for clients and family members. Family members also frequently discussed the effects of stigma and lack of understanding as barriers to support in the wider community. These themes were acknowledged, discussed, and confirmed by caseworkers. Family members and caseworkers also addressed specific gaps in care and potential ways to provide additional supports to clients and family members.

### The emergence of potential concepts

The final step of the analysis for this study required examining the connections between the categories developed from both interviews and focus groups until overarching narrative concepts emerged. The concept uniting the data that emerged was that of the struggle clients and their families faced to gain access to healthcare services. These struggles fell into two major sub-themes: struggles to get information and early assessment during the prodromal stage of psychosis, and struggles to get timely and adequate diagnosis, treatment, and support both in the hospital and the community. As the illness progressed, these clients and family members would struggle to differing degrees with these challenges. These sub-themes are elaborated below, including the input of caseworkers.

### Struggles during childhood and the prodromal stage of psychosis

A repeated element across many interviews with family members was their memories of their young child having initially typical development, being sociable, outgoing and active in extracurricular activities. Eight of the thirteen clients showed no signs of struggle until prodromal symptoms first appeared during junior high or high school, causing the trajectory of their academic route to alter quickly and dramatically. The other five clients had a similar early progression, with the exception of some difficulties in concentrating, attention, and performance apparent in earlier childhood. Of the five clients, three were diagnosed and being treated for ADHD before the end of elementary school. The other two were diagnosed with autism spectrum disorder and dyslexia, respectively, in early adulthood. Two of these five clients had a full assessment performed by their school and were granted individualized supports and accommodations. One of these clients refused to use the supports granted by the school as they believed the supports singled them out from other students.

As these students moved into the early prodromal stage, they were often labelled as having behaviour problems, being lazy, or disinterested in school. Eight family members recalled the point where the behaviours of their child caused them to want their child assessed by a health professional, most often the family physician. These interactions were invariably disappointing and frustrating for all involved as the family physician tried various medications to treat more common psychiatric conditions, often ADHD and anxiety. Five family members recalled that clients would take the medications prescribed but quickly discontinued them when they failed to help or when adverse effects developed. These clients then lost faith in the family doctor and refused to return. Three clients also reported this experience. Two clients were referred to a child and adolescent psychiatrist, which resulted in a similar experience.

As the prodromal stage of psychosis became more pronounced, the clients started to isolate. Family members recalled becoming increasingly distressed by the client but feeling unable to help. This often started their journey of struggling to find information. As one family member stated,

“We were often given pamphlets on depression, suicide, mental health services, phone numbers, agencies, organizations, and so on to get a sympathetic ear. That was it, and that was great. That was well-intentioned, it was well-meaning, but it wasn’t what we needed.”

Meanwhile, clients often struggled to attend school, and six of thirteen left school without graduating. Of those six, three returned as adults to graduate with their high school diplomas. One client and two family members of clients recalled the need to enforce school attendance to ensure that the clients graduated. During the school-aged years, clients and family members identified a lack of trained educational staff to recognize potential psychosis and felt that changes could be made in how services are provided to children, especially streamlining the referral process from schools to the mental health system. They added that there should be a mechanism for clients to self-refer for mental health services.

Substance misuse surfaced as a prominent theme amongst the thirteen families, with participants stressing that the frequency and potency of this use escalated rapidly. Alcohol (eleven out of thirteen) and cannabis (ten out of thirteen) were the most common substances reported as misused, with cocaine (three out of thirteen) also appearing multiple times. Three family members spoke about suspicions that a client was misusing recreational substances, but were unable to confirm their suspicions or name specific substances. While substance misuse is not atypical for this age group, what was unique to this group was the dramatic and alarming escalation in usage. The use of cannabis often started slowly and then increased in both frequency and potency until the client was a frequent user of high-potency cannabis. Other recreational substances reported as misused by clients included prescription medications, MDMA, LSD, psilocybin, and heroin. Family members felt that the sudden change in academic progress, coupled with the dramatic escalation of substance misuse, should have been an alarm for schools. They discussed the struggle to have their concerns acknowledged by school staff.

The caseworkers recognized many of these statements made by clients and families as aligning with what they see in their practice. One caseworker spoke specifically about the harm done by labels that a client may receive during the early days of schooling, that being called ungrateful, lazy, or unmotivated creates a label for impressionable children, which they internalize. Two caseworkers also reported that stigma and “labelling” extend to physicians practicing outside psychiatry. Their experience was that youth with substance misuse issues received different treatment from physicians who were not psychiatrists compared to youth without such issues: “Now they’re not seen as a child who’s experiencing behavioural trouble or psychological trouble. Now it’s well, you’re a drug addict, and we need to focus on that before we can focus on your mental health”. While the caseworkers recognized the prevalence of stigma and discrimination, they felt that it did not prevent clients from seeking treatment. They did identify that community stigma has two major effects on clients and their families. First, clients and families are more isolated, often feeling unable to talk candidly about their struggles with family and friends. Second, there is less understanding and empathy than for those living with physical conditions. This severely limits client and family member access to large advocacy groups with widespread public support.

Caseworkers reported hearing from clients that they lose interest in school because of a lack of ability to focus and engage. Family members voiced concern for the client’s struggles with hygiene, cognitive focus, becoming easily agitated, and isolation. Caseworkers also spoke about the efforts families take to get a client assessed by a family physician or other specialists, stressing that many in the province do not have access to a family physician. They recognized that treating youth is a challenging proposition and often takes an extended period of time. They added, however, that when misdiagnosed and prescribed ineffective medications, youth become frustrated with both the effects of the medications and the belief that their own concerns are not being taken into consideration. These were reported as major reasons why clients choose to discontinue medications and physician visits, leading to substance misuse. One caseworker, whose main practice is with school-aged children, commented on the quick and dramatic escalation in substance misuse: “everything is on fast-forward, everything is sped up”. The caseworker continued by saying that not only has the rate of misuse increased, but the age of first use has decreased, often beginning in junior high. Additionally, the substances used by school-aged children are becoming increasingly dangerous, with cocaine and MDMA gaining prevalence and a smaller but increasing number of youth using LSD and crack cocaine. Caseworkers expressed the concern that many physicians view substance misuse as a separate issue that must be resolved before a client can be treated for their psychosis, rather than an integrated issue which directly interacts with the symptoms of the client’s psychotic disorder.

### Struggles to get the appropriate diagnosis and treatment

By late adolescence, the symptoms of clients were so severe that families were much more active in seeking medical support. However, nearly all participants described a long interval between the onset of discernible psychotic symptoms and obtaining a formal diagnosis of a psychotic disorder, often lasting twelve to eighteen months. Most acknowledged that substance misuse complicated this process.

Difficulty in having a client admitted to an adult psychiatric facility also surfaced in several interviews with family members. They expressed frustration that clients were turned away from the hospital assessment unit without treatment, even when presenting with severe symptoms, often being brought back days later by the police and admitted involuntarily. Several family members reported that their child’s first hospitalization resulted in a diagnosis of substance-induced psychosis. The client was then discharged into the care of the family with no further discussion about how the family should proceed to support the client, lengthening the time from symptom onset to diagnosis. One parent expressed understanding that psychiatrists are hesitant to diagnose a psychotic illness, but they felt deeply frustrated at the lag between symptoms and diagnosis: “And of course, they’re not going to be very quick to diagnose something as severe as schizophrenia, I understand that. But you know, it was… you’re pretty well left out to the wolves after that first visit, right?” Another parent recalled having to follow the staff psychiatrist on their rounds to speak with them after their child was hospitalized: “I spent a few weeks in hospital just pretty much chasing people around on rounds in the morning. Well, what are we gonna do, like, what’re we gonna try to put him on, and things like that.” As traumatic as hospitalization can be, family members reported that it was often the only route to the client receiving a diagnosis or treatment.

The services provided by the police and mobile crisis units in dealing with clients also surfaced, with perspectives often differing. Some parents voiced positive experiences and were appreciative of the service, even though they felt an obligation to do what one parent referred to as the “nasty work” of calling the police when their child became dangerous to themselves or others. Others revealed unresolved frustration at the response of the police, who they felt often lacked sufficient training to handle mental health calls and seldom attempted to de-escalate tense situations.

Another issue for many family members was that they were excluded from the adult health system, and they were helpless in watching their child struggle without appropriate services. They appreciated the difference between child and adult healthcare systems and the concern for confidentiality and adult autonomy, but felt their inclusion would have expedited the process and lessened their child’s struggles. One family member stated that these policies were indeed well-intentioned but did not consider the realities of a client living with the effects of a psychotic illness. Another family member stated, “I can say with all authority that it’s been the hardest thing in the world to try and get him some help”. They felt that the health system needs to embrace the family in the decision-making process for clients to a greater degree. Family members have knowledge and insights into client history and symptoms that mental health staff do not possess, which they feel is vital information. As an example, a family history of mood, anxiety, and/or substance use disorders was a recurring topic in interviews, with two families having close relatives diagnosed with a psychotic disorder. Many family members discussed the need for the mental health system to treat the family as a whole, not just the client. Three families were able to either send clients to other provinces in Canada for care or move permanently to other provinces. Participants were unanimous that decreasing the time a client spends in untreated psychosis would improve health outcomes. They believed that the system of psychiatric assessment and admission should be altered, with easier access to psychiatric beds and faster access to psychiatrists.

Caseworkers recognized these statements relating to access to services and supports. One caseworker added that often, when clients are newly hospitalized, they do not know why they are inpatients and have not yet been told their diagnosis or had it accurately explained. Another caseworker stated that the staff in adult psychiatry will not make a diagnosis of a psychotic disorder if the client is under the effects of recreational substances, preferring to offer a diagnosis of substance-induced psychosis. Several caseworkers stated they understood this hesitancy, stressing that clinicians will only diagnose a psychotic disorder when they feel that a client has crossed a clinical threshold, and no other disorder could be responsible. Caseworkers commented on the diversity of family experiences with the psychiatric assessment units and crisis response teams, particularly with getting support at the assessment unit. They stated that the mobile crisis response teams units are effective, but say that the criteria for detaining clients under the Mental Health Act are so stringent that police seldom use it as a means of detaining a client. This results in family members having to decide if they wish to have a client released immediately back into their care or detained on criminal charges.

### Struggles with medication management

In addition to struggles with ineffective medications before diagnosis, struggles with accessing appropriate medications post-diagnosis surfaced. These struggles can be divided into three sources: getting a medication adjusted, getting a prescription filled, and experiencing adverse side effects. First, participants identified an issue in getting an appointment with their psychiatrist to review their medications. Family physicians stated that psychosis management was out of their scope of practice, yet getting an appointment with a psychiatrist was challenging. This was exacerbated by the reality that it often requires several changes in medication to find the appropriate medication and dose to manage the client’s symptoms. During this wait time, clients and family members were forced to contend with the adverse effects of the medications. Families living in rural areas were particularly affected by this, where wait times to see a psychiatrist can exceed six months.

Second, once a medication is prescribed, there can be challenges in getting it filled. Clients who live in rural areas reported delays in getting their vital medications shipped to their local pharmacies. Insurance coverage for medications, including the provincial drug plan, also surfaced. Several participants discovered that their insurance would not cover their prescribed medication, leaving them the choice of paying themselves or risking a relapse. One parent recounted: “I got into a full-blown argument at my work one day in a parking lot with someone who said no, you know, you’re not covered. Because he takes two antipsychotics, he’s not covered.” This situation was more common for prescriptions of long-acting injectable (LAI) medications, often prescribed by psychiatrists when a client has difficulty taking oral medications daily. Another issue reported was the stipulation that two antipsychotic prescriptions could not be covered at once. Family members described these issues as being exceptionally frustrating to watch and a major obstacle to a client’s stabilization and recovery.

Third, the adverse effects of antipsychotic medications surfaced, especially heavy sedation and conspicuous weight gain. Other adverse effects included vomiting, heart issues, and difficulty urinating. The feeling of being overly sedated was often named as a reason for discontinuing antipsychotics in favour of attempting to self-medicate with recreational substances. Family members reported worrying about how heavy sedation affected clients and their ability to be employed. One parent worried that their child’s sedation was so severe that he might not wake up if his smoke detector activated during the night: “Like, it’s actually worrisome because if he had a smoke detector by his head, he would not hear it.” Another parent living in a rural area of the province explained the difficulty of finding health specialists in their area to address the adverse effects caused by their child’s medication.

The complications of accessing appropriate medications, adverse effects, and their resultant impact on chronic substance misuse were recognized by the caseworkers. They identified these issues as a leading cause of relapse in substance misuse, with clients struggling with compliance and returning to using substances after they were unable to tolerate antipsychotics. They added that difficulties in having prescribed medications covered by the provincial drug program are a widespread issue that has a disproportionate impact on these clients.

### Struggles with family relationships

The relationship between family members and clients repeatedly surfaced as a topic throughout the interviews. All participants were in various stages of the journey through a psychotic illness, with some families having a member with a new diagnosis, while others were diagnosed years ago. Family members often spoke positively of finding a peer support network with specific experience in supporting clients with psychotic illness and their families. They credited these groups with giving them the tools to better understand client behaviours and constructively interact with them. Despite this, many family members reported struggling in their relationships with clients. An absence of the client having a clear understanding of their symptoms or acceptance that their behaviours and beliefs were out of the ordinary seemed to fuel this. Client anger and frustration directed at family members for their hospitalization was also common. These emotions would often resolve, but in other cases, the client remained upset and mistrustful of the family members.

Many families discussed their own journey as their child progressed through their illness. Two elements of these conversations appeared across many interviews. The first was the independent search for information on schizophrenia and related disorders, as well as available treatment methods. Family members identified a lack of accurate general knowledge about psychosis and psychotic illness in the general public. Many recalled that the media was often the only source of information they had. One parent recounted their actions the day after their son was diagnosed:

“I went to the bookstore to try to, the day after [the diagnosis], actually, that I got out there to try to find some kind of information, anything at all, on schizophrenia. Because I was just lost, I didn’t know what to do for him, and I didn’t know, you know what, what to suggest.”

The second element was a kind of grieving process that was carefully described by many family members. Family members would often fondly recall how a client presented during their childhood in comparison to how they presented as adults. Their emotions stemmed from the acceptance process and the slow realization that their child’s life would be different from what was envisioned.

Interviews with three family members discussed the involvement of the client with the criminal justice system. Family members commented on the prevalence of severe and persistent mental health issues in the criminal justice system, feeling that they would be better treated by the mental health system. A criminal record was discussed as a point of trauma for the clients attempting to reintegrate into society with the added stigma of criminality. Those families voiced a need for trauma support in coping with their experiences with the justice system.

Caseworkers recognized the strain put on family relationships when a child has a psychotic illness. One caseworker specifically commented on how parents often would recount in detail how different their children were before the onset of psychotic symptoms. The difficulty family members have in convincing their loved one to seek professional mental health care was identified by several caseworkers as a significant stressor. One caseworker also stated that several clients they supported opted for the criminal courts rather than the mental health courts to avoid being officially labelled with a psychotic illness.

### Struggles with community supports

Accessing community supports and services was another prominent topic, especially in rural areas. A parent living in a rural area expressed deep frustration that services for their family member were only available in the city, resulting in them having to stay in the city. Housing was a gap that surfaced with several families, with periods of homelessness being common for many clients. Relapses and hospitalizations often caused housing instability, and several participants reported that landlords discriminate against clients with psychotic illness. Discrimination continued to be a subject of many conversations, extending to employment. Clients and families reported striving toward steady employment as a symbol of client independence. Family members often reported worrying that employers would not hire a person with a psychotic illness.

Another break in services was access to appropriate mental health counselling. Family members reported that hospitals discharged clients as quickly as possible, regardless of whether counselling supports were in place or if they had other services or supports to help with reintegration into the community. Family members often discussed struggles with the community-based teams dealing with clients living with psychosis, describing those teams as focused solely on ensuring a client is taking their medications regularly and not a threat to themselves or others. Other areas of required services included applying for employment insurance and returning to post-secondary education. One parent spoke about attending a meeting with their child and the accessibility coordinator of a large post-secondary institution:

“But you’re dealing with someone who’s very well educated in what they can offer but not educated into disabilities and how you deal with different people. And I think that’s really important when you’re going to school, to have somebody to be able to, you know, understand that you need to change the way you talk to people and, you know, like, the conversation that should have taken 10 minutes took 40 minutes.”

Existing services in the community were described by one family member as being “dots” of support, disconnected and often difficult to access. Multiple family members called for an overarching organization to coordinate services in consultation with the mental health system to prevent the struggle with access.

Caseworkers recognized these gaps in service as congruous with their caseloads. They called for psychiatric assessment units to create a wraparound service to provide community-based treatment plans for individuals experiencing distress. Caseworkers also spoke about their efforts to stop evictions as well as the isolation and loneliness observed in clients, especially among older clients, resulting in an increased risk of substance misuse and suicide. Multiple caseworkers spoke about the need for enough resources to meet demand, as well as a single organization to coordinate programs. One caseworker mentioned that those with severe and persistent mental illness and little community support often prefer to stay in a psychiatric hospital where they feel safer and have less judgment.

## Discussion

### Identifying emerging patterns in early childhood and the psychosis prodrome

Specific symptoms predicting clinical high risk (CHR) for psychosis are well studied and include attenuated psychotic symptoms, presence of psychological stressors, family history of a psychotic disorder, and male sex [35–37]. Research is now examining the area of brain imaging and proteins found in blood samples as methods for the early diagnosis of psychotic illness [38,39]. While this is exciting, there will be a waiting period before this knowledge may be translated into clinical practice. Additionally, the heterogeneity of individuals in terms of clinical presentations and biomarkers complicates the research being conducted [40].

Early recognition of youth who are at a CHR for a psychotic illness is vital. While only a portion of youth with CHR status will progress to being diagnosed with a psychotic disorder, those who are not diagnosed will continue struggling with mental illness for years into adulthood [41]. This research project describes the trajectory that clients and family members travel on their journeys after the symptoms of psychosis appear. It also details the struggles they must contend with in accessing treatment.

### Identifying gaps in services for psychotic illness

The third focus of this research was to identify gaps in services. Three distinct areas emerged: schools’ awareness of psychosis and ability to identify warning signs, the mental healthcare system’s ability to provide timely and effective treatment, and community supports. Identified gaps in supports and the suggestions provided by the participants were often in line with existing literature. Participants consistently described gaps across multiple services, including education, healthcare, pharmacy, justice, housing, and social services.

This study identified a need for a system of early alarms, especially when the apparent trajectory of a youth’s life shifts and is coupled with substance misuse. The literature in education identifies these roles as being essential in fostering student well-being [42–44]. Mental illness in Newfoundland and Labrador has traditionally been stigmatized, and those living with mental illness have been underserved and ignored. This has begun to change in recent years, with an All-Party Committee being convened in 2015 [45] to examine the state of mental health services in the province. It released a report in 2017 outlining 54 recommendations as part of a five-year action plan to improve mental health and addiction services in the province. The NL Health Accord [6], published in 2022, also identified mental health as an area of concern for youth. Most recently, an All-Party Committee [46] released a report in the summer of 2025, which acknowledged the progress that had been made but also contained targeted recommendations to improve the current level of services that are available to clients and families.

Calls by family members in this province to be involved in client treatment are supported by the literature [36], which indicates increases in the accuracy and speed of diagnosis when family members are part of the process. Finland’s open dialogue approach emphasizes a flexible strategy that incorporates several vital elements: a rapid response to acute psychosis, family-centred therapy as well as individual therapy, and interactions between the treatment team, the client and their entire social network [47]. Research is ongoing on how this approach can be adapted to other settings [48,49]. A similar approach to management is the wraparound care model pioneered in the 1980s by Stroul and Friedman [50] and still used today to support youth with complex needs [51,52]. Finally, financial losses caused by psychosis can be costly, with a client’s first year after diagnosis costing hospitals 24,000 US dollars on average [53]. Community-based supports, such as those for employment and housing, are not included in this number. These indirect costs can account for up to 70 percent of the total financial losses inflicted by psychotic illnesses [54].

### Study strengths and limitations and possibilities for future research

The voices of this cohort of participants offer a unique look into the lived experience of clients and family members, along with the additional insights of caseworkers. While the more vulnerable and stigmatized clients may have been excluded from participating, the perspectives of case workers helped to triangulate the emergent themes against a much larger client base, over time and geography. The information presented in this study relates only to the province of Newfoundland and Labrador. Future studies comparing the lived experiences of clients and family members across other regions would be a valuable addition to the literature.

### Implications on practice

The results of this study have implications for clinicians, caseworkers, school staff, and policymakers. The research described here serves four important purposes. First, it gives an insight into the beginning stages of psychosis as it appears in youth and how the illness progresses into adolescence and adulthood. Second, it equips those interacting with clients and family members with the knowledge to be able to recognize patterns of symptoms, behaviours, and access-seeking that may predict the onset of psychosis, and to provide appropriate supports. Third, it identified gaps in services as experienced by clients and family members. Fourth, this study presents recommendations of those with lived experience and their caseworkers on how the current system can be changed to improve services and increase the likelihood of positive health outcomes.

### Developing concepts into theory

When assessing how the concepts described in this study can contribute to a grounded theory, we must first return to the four criteria developed by Charmaz [29,30]. The four criteria of credibility, originality, resonance, and usefulness identify the presence of quality and rigour in grounded theory research. First, the credibility of this study was upheld by two methods: collecting data using theoretical sampling to ensure data saturation and using memos to consistently reflect on innate perceptions and biases. Second, this study demonstrates originality by being the first in the province of Newfoundland and Labrador to conduct research into psychotic disorders as they develop, are diagnosed, and managed. The researchers developed a set of interview questions and focus group topics specifically for this study to uncover different perspectives. Third, the perspectives of clients, family members, and caseworkers were integrated into this study and were directly related to the reader to achieve resonance. Finally, the usefulness of this study is demonstrated by the fact that it: 1) presents the lived experiences of a vulnerable group of people, 2) prompts the conducting of further research in the area, and 3) provides an incentive to strengthen existing policies and programs as well as develop new ones.

This research used the experiences of clients, family members, and caseworkers to produce categories, which were refined into concepts that contribute to the grounded theory describing the experiences of this population. First, the concept of struggling with the first symptoms of the psychosis prodrome in school and across social settings appeared from the theoretical categories derived from the data. Second, the data drawn from the research support the concept of struggling to receive a formal diagnosis, as well as consistent and appropriate treatment, including the management of symptoms and adverse effects caused by prescribed medications. The third concept that emerged from the data analysis was the tangible effect of significant gaps in service to the clients and the family members. These structural-level gaps in service were felt across multiple settings, including schools, hospitals, and the community. The result of these gaps in services was demonstrated by: 1) the increase of stress on clients and the burden of care on family members, causing additional trauma, 2) the isolation of both groups from the greater community and 3) the lack of access to existing services that have no clear and accessible care pathway.

## Conclusion

Psychosis and psychotic disorders are a devastating reality for clients and family members. The stigma attached to psychosis and psychotic disorders has meant that for many, they are unable to get the support that they require to face the consequences that come with a severe and persistent mental illness. This study is an examination of the pathways to care that clients and family members pursue to obtain access to information, diagnosis, and treatment. It adds the experiences and knowledge of caseworkers to enrich those experiences, offering insight into the experience of highly vulnerable clients and their families. It is presented as a tool to guide clinicians, caseworkers, school staff, and policymakers. Its purpose is to improve the services provided to clients and families and optimize their futures.

## Supporting information

S1 File Interview guide for clients and for family members. (DOCX)

S2 File Focus group guide for caseworkers. (DOCX)
