# Supplementary material for: Factors that affect pathways to care for youth with psychotic disorders in Newfoundland and Labrador, Canada: A qualitative study
Source: PLOS Ment Health. 2026 May 14;3(5):e0000612. doi: 10.1371/journal.pmen.0000612 (PMC13175373; doi:10.1371/journal.pmen.0000612)
Supplement: S1 File — (DOCX) [file pmen.0000612.s001.docx]

**S1 File. Interview guides for clients and for family members.**

**Topic guide for client in-depth interview**

Introduction:

Thank you for agreeing to take part in this interview. Your answers will be helpful in understanding the way people access care for psychotic illnesses.

Before we begin the interview, I would like to clarify a few things:

- This interview will be used for research purposes. No identifying information will be included in the final study report.
- During this interview, you can take a break at any time you want. You may also choose to not answer any question. You also can stop the interview at any time.
- After the interview, you have up to six weeks to request that your interview not be included. In which case, all data collected from you will be destroyed.
- After five years, all the data from the study will be destroyed. This is a Memorial University rule.
- Earlier, you told me you do/do not wish this interview to be recorded. Is that still what you want?
- You can think of it as more of an informal conversation. I would like to hear your story in your own words. I ask the same questions to everyone. I do this to help me understand and find any themes and shared experiences.
- If you have any questions or comments about this interview that you do not want to speak to me about, you can contact my supervisor, Dr. Gerald Mugford. You can also contact the Health Research Ethics Board. I will put their contact information in the chat box/ Here is the sheet with their contact information.

Are you ready to begin?

******(**Note to reader:** the subsequent interview questions are intended to flow in a conversational manner. The indicated probe questions will only be asked if the interviewer deems it to be necessary to collect relevant information)******

1. **Demographic information**

Alright, let’s start off with asking some basic information:

1. How old are you?
2. What’s your highest level of education?
3. Are you currently working? How long have you been [*status*]?
4. Where are you living now? How long have you been [*arrangement*]?
5. What medical professionals are you seeing now? How long have they been following you?
6. Have you seen professionals in the past that you’re not seeing now? If so, which ones? How long did you see them for? Do you know what age you were?
7. **Educational information**

Let’s start by focusing on your experiences in school. Can you describe those years for me?

Clarify

- favourite teachers
- favourite subjects
- extracurriculars
- academic struggles
- attendance patterns
- level of education completed
- types of supports received in schools
- individualized plans created
- referrals to other professionals
- school counsellors used
- post-secondary experiences

1. **Community information**

Let’s focus on your social experiences. Can you tell me a little bit about that?

Clarify

- Community supports while growing up (family, friends, etc.)
- Current community supports (family, friends, etc.)
- Use of alcohol/tobacco
- Use of recreational drugs
- Interactions with the police

1. **Medical information**

Let’s shift focus and talk about medical interventions. Can you describe what accessing medical supports has been like for you? What helped, what was challenging for you?

Clarify

- when referred for psychiatric assessment(s)
- waitlist to see professionals
- professionals seen (counsellor, psychologist, MH nurse, GP, Psychiatrist, etc.)
- who’s following you now
- meds currently taking, do they help, are there side effects
- meds previously taken, how long did you take them, when was this
- current diagnosis/es, is/are they accurate
- transition from child to adult services
- family history of psychiatric illness and/or substance abuse
- emergency room visits

1. **Reflection**
2. Looking back, is there anything more you wish had been done to help you?

- school system
- health care system
- justice system (if applicable)

1. What do you feel like is your current state of wellbeing?
2. What do you feel you need right now, if anything, to improve your wellbeing?

3. This study is looking to understand the experiences of people with your history, in the

hope of helping others. Do you have any final thoughts or comments that you would

like to share?

- any topic that did not cover what you think should have been covered?

Thank you for your time.

**Are you comfortable with me interviewing your parent to help understand your medical and educational history?**

**Topic guide for family member in-depth interview**

Introduction:

Thank you for agreeing to take part in this interview. Your answers will help understand how people access care for psychotic illnesses.

Before we begin the interview, I would like to clarify a few things:

- This interview will be used for research purposes. No identifying information will be included in the final study report.
- During this interview, you can take a break at any time you want. You may also choose to not answer any question. You also can stop the interview at any time.
- After the interview, you have up to six weeks to request that your interview not be included. In which case, all data collected from you will be destroyed.
- After five years, all the data from the study will be destroyed. This is a Memorial University rule.
- Earlier, you told me you do/do not wish this interview to be recorded. Is that still what you want?
- You can think of it as more of an informal conversation. I would like to hear your story in your own words. I ask the same questions to everyone. I do this to help me understand and find any themes and shared experiences.
- If you have any questions or comments about this interview that you do not want to speak to me about, you can contact my supervisor, Dr. Gerald Mugford. You can also contact or the Health Research Ethics Board. I will put their contact information in the chat box/ Here is the sheet with their contact information.

Are you ready to begin??

******(**Note to reader:** the subsequent interview questions are intended to flow in a conversational manner. The indicated probe questions will only be asked if the interviewer deems it to be necessary to collect relevant information)******

I’ve met with [*client’s name*] and we had a good conversation. I’m not going to ask you the same questions, but rather collect your memories of their experiences. I may also be asking some clarification questions if any come up.

1. **Background Information**

Let’s begin by you telling me about your perspective of [*client’s name*]’s struggles with mental health.

Clarify:

- When did they start?
- How did they progress?
- What was your experience with this?
- Parents’ current relationship with [*child’s name*]?

1. **Education information**

Let’s focus in on [*client’s name*]’s school experience. How did you feel that impacted [*client’s name]*? Can you describe those years for me?

Clarify

- supports that were needed in school
- type of supports
- accessing those supports
- counselling support
- transition from school to post-secondary/work
- individualized support plans
- attendance
- assessments made by school staff

1. **Community information**

Let’s focus in on [*client’s name*]’s social experiences and how you felt that was impacted. Can you tell me a little bit about that?

Clarify

- community supports while growing up (family, friends, non-profits, etc.)
- current community supports (family, friends, non-profits, etc.)
- any difficulties that [*child’s name*] experienced in the community (drugs, police interactions, etc.)

1. **Medical information**

Now, let’s focus on medical interventions. Can you describe what accessing medical supports has been like for [*client’s name*]?

Clarify

- to whom referrals were made
- when were they made
- accessing supports
- transition from child to adult health
- frustrations or challenges encountered
- family history of learning or mental health issues
- substance abuse
- history of medications taken
- which medications helped, which didn’t help
- exact history and chronology of diagnosis

**E. Reflection**

1. Looking back, do you think more could have been done to help [*client’s name*]?

- school system
- health care system
- justice system (if applicable)

1. This study is looking to understand the experiences of people with [*child’s name*]’s history, in the hope of helping others. Do you have any final thoughts or comments that you would like to share?

- any topic we did not cover that you think should discuss

Thank you for your time.
