# Supplementary material for: Factors that affect pathways to care for youth with psychotic disorders in Newfoundland and Labrador, Canada: A qualitative study
Source: PLOS Ment Health. 2026 May 14;3(5):e0000612. doi: 10.1371/journal.pmen.0000612 (PMC13175373; doi:10.1371/journal.pmen.0000612)
Supplement: S2 File — (DOCX) [file pmen.0000612.s002.docx]

**S2 File. Focus group guide for caseworkers.**

**Focus group guide for caseworkers**

*Introduction to Focus Group*:

- Thank you for being part of today’s focus group, your participation is deeply appreciated.
- As outlined in the consent form, this focus group may be recorded. You have the right to turn off your camera at any point.
- No information that can be used to identify you will be included in any final documents produced by this research. Given the relatively small number of caseworkers in this area, however, confidentiality cannot be guaranteed, as your participation may be assumed.
- You are kindly asked to refrain from identifying any individual client during the focus group. If you do accidentally give identifying information, it will not be included in the transcript of the focus group.
- Are there any questions before we begin?

As you know, my study focuses on one research question: what are the variables and indicators that affect pathways to care for youth in Newfoundland and Labrador accessing treatment for psychosis and psychotic illness? These variables and indicators may be present before or after the onset of frank psychosis. However, their identification will contribute to strengthening early intervention and treatment and lessen the duration of untreated psychosis in this vulnerable population.

Today, I am seeking your perspective on themes that emerged from the interviews that I’ve conducted with these clients and their family members. I’m looking for your insights into how common these themes are, given your unique position of providing services to clients and families living with psychosis and psychotic illness. Your participation today will be used to help improve current pathways to care models in the province for those accessing services for psychosis and psychotic illness.

*Topics for Discussion*:

1. a. Many clients reported doing well during the earlier grades, and even into the junior high years, but then starting to struggle academically in high school. Several reported being identified with learning disabilities and anxiety (diagnosed in high school), and some were diagnosed with ADHD (although medication often did not help and was eventually discontinued). Is this typical of your clients? Has it changed over the years? What are your thoughts on how it may affect access to care?

b. These struggles in high school led to these individuals falling behind and becoming disengaged, with many failing to complete high school. Several reported being described as lazy. Is this pattern typical in your caseload? Has it changed over the years? What are your thoughts on how it may affect access to care? Are there early alerts that could be identified during the school years for this cohort? Or…In your experience, should this dramatic change in their academic pathway and progress be an early alert?

1. These clients were often followed by a family doctor who was described as becoming increasingly frustrated by their struggles to help stabilize their needs, despite sincere efforts. This was often exacerbated by the clients moving into adulthood when the parent could no longer accompany them to medical appointments, and/or the client missing appointments. Is this pattern typical of your clients? What are your thoughts on how it may affect access to care? How do you think family members might become more integrated into pathways to care for adult patients?
2. One theme that emerged was the use of recreational drugs, beginning in early high school but escalating very quickly, much faster than that of their peers. Is this quick escalation in recreational drug use typical of your clients? Has it changed over the years? How do you think it has impacted their access to care?
3. Families reported dealing with behaviours that were so severe that they felt the need to call the police, yet hesitated to do so for fear of the police and possible outcomes. Those who did call the police reported regret for doing so and described the experience as traumatizing. Is this a common theme among your families? What are your thoughts on how it may affect access to care?
4. Parents of clients report that the client struggled with symptomology often for years before eventually finding support. This process can take even longer in rural communities. Many clients and families reported several hospitalizations before being diagnosed with a psychotic illness and receiving appropriate care. Many family members report that it was and remains difficult to get healthcare workers to listen to them. Is this typical of your clients? What are your thoughts on how it may affect access to care? Do you think there might be early alerts for a health team that could expedite access to care during these hospitalizations?
5. Once a diagnosis was made, access to appropriate medications also arose as a common theme across interviews. This included accessing a clinician who would prescribe appropriate medications, as well as getting prescriptions filled in a timely manner. In your caseload, are you seeing these issues being raised by clients and families? Has it changed over the years? What are your thoughts on how it may affect access to care?
6. The adverse effects of antipsychotic medication, especially heavy tranquillization and conspicuous weight gain constituted a frequent theme in interviews with clients and family members. These interviews also identified a lack of concern or support from health care in dealing with this. Is this common to your caseload? What are your thoughts on how it may affect access to care?
7. Stigma and a lack of support and understanding in the community also surfaced as a hindrance to accessing care. Family members commented that their loved one can find it difficult to access community supports even when they’re well. They also have difficulty accessing all types of medical care due to the symptoms of their psychosis. Is this a common issue in your case load? Has it changed over the years? What are your thoughts on how it may affect access to care?
